# Report of One Hundred and Eleven Cases of Cancer Treated with the X-Ray with Comments Thereon

**Published:** 1907-12

**Authors:** Ennion G. Williams

**Affiliations:** Professor of Pathology, Medical College of Virginia, Richmond, Virginia


### "REPORT OF ONE HUNDRED AND ELEVEN CASES OF CANCER TREATED WITH THE X-RAY WITH COMMENTS THEREON."

BY DR. ENNION G. WILLIAMS, PROFESSOR OF PATHOLOGY, MEDICAL COLLEGE OF VIRGINIA, RICHMOND, VIRGINIA.

Abstract of paper read before the American Roentgen Society, Cincinnati. October 1907,

The cases were unselected and represented the work as it occurred in his X-ray practice. He emphasizes the vast difference between the clinical nature of different cases of cancer, and states that it is only possible to approach an estimate of the value of the ray, by studying a large number of cases, divided into classes, which are more or less similar and consequently susceptible of comparison.

The cases are classified in six tables, which show the date of treatment, age, sex, location and extent of growth, number of exposures, duration of treatment, the result, and remarks concerning points of special interest.

The FIRST CLASS includes those growths on the skin, advanced beyond a keratosis, having a thick scale covering an ulcerated area of long standing, with the history of growing more rapidly of late and also those growths with elevated margins and ulcerated centers, growing steadily, having started usually from a mole or papilloma.

Of the fifty-three (53) cases, fifty-two (52) healed. In the case not healed the treatment was interupted by an attack of pneumonia. The growth was afterwards excised. In four of the fifty-two cases, there was a recurrence which healed again with the X-ray. The first case was healed four and a half years ago. The average number of exposures was sixteen (16) and the average duration of treatment was five (5) weeks.

The SECOND CLASS includes those growths which originally began as those of the First Class, but have reached a more advanced stage and invaded the subcutaneous tissues, either leaving in their wake a deep ulcerated and sloughing area, or by the rapid proliferation of malignant cells, have given rise to a large protuberant or rose-like mass.

Of the seventeen (17) cases, eleven (11) were healed, in six (6) of which there has been no recurrence or evidence of malignancy to the present time; in the remaining five (5) there was a recurrence. Four cases were improved. Two (2) were unimproved.

The average number of exposures was forty-nine (49). The average duration of treatment was five (5) months.

The THIRD CLASS includes the extensive recurrent and metastatic growths, in the seep structures. The original growth may or may not have been on the skin.

Of the nine (9) cases reported, in two (2) there was an apparent disappearance of the growth. One has remainel healed twenty (20) months. The case was a recurrent growth after paste treatment, and involved the nasal and frontal bones. In the other case there was a recurrence.

Two cases were temporarily improved, that is, the pain was relieved, and there was a decrease in the size of the growth. Five cases were unimproved.

The FOURTH CLASS includes carcinomas on mucous membranes.

Of ten (10) cases, five (5) were on the lower lip. Four (4) of these were temporarily improved, but not healed, and were afterwards excised. One case was unimproved. Excision is now advised for growths on the lip.

In three (3) cases there was extensive involvement of the tongue, floor of the mouth and glands of the neck. They were unimproved.

One case of carcinoma of both tonsillar regions was temporarily benefitted, by the relief of pain and diminution in the size of the growth on one side. The growth later progressed and terminated fatally.

The FIFTH CLASS includes primary cacinonas of the breast.

There were three (3) cases,—one was given eight (8) exposures in eight days. A few days later a radical operation was performed. Microscopic examination of the tissue, removed, showed disintegration of cells in many alveoli. Patient later died of recurrence.

Second case, a tumor in upper outer quadrant of left breast, supposedly malignant. After ninety (90) exposures in ten (10) months, the tumor had shrunken to a small indurated mass. There has been no reviving of the growth, now after three (3) years.

The third case was a carcinoma, beginning on the side of the breast. After fifteen (15) exposures, extending over one month, a dermatitis was reproduced, which subsided, accompanied by the disappearance of the growth. Twenty-six (26) months later it returned, was treated by ray and disappeared again.

The SIXTH CLASS includes the recurrent carcinomas of the breast.

Of fifteen (15) cases treated, three (3) were unimproved. Seven (7) showed great improvement by the reduction of growth, and the relief of pain. In five (5) there was complete disappearance of all evidence of disease.

In three (3) of these five (5) the disease reappeared a year later.

In the other two (2) there has been no recurrence to the present time, two and one-half (2½) years and four months afterwards respectively.

The recurrence of a malignant growth is dependent upon whether or not all the malignant cells have been destroyed. If the ray has destroyed so many of the cells that the growth has been reduced to a mass invisible to the eye, the ray will undoubtedly destroy the remaining cells, if its distinctive influence be continued.

Many of the superficial growths healed without producing any reddening of the surrounding tissues.

Healing seemed to take place better when the growths were exposed to the air and sunlight, than when covered with dressings and ointments.

The apparatus used was two static machines and three coils, with mechanical, mercury and electrolytic interruptors. The distance of the anticathode from the surface, varied from six (6) to ten (10) inches in deep cases. The duration of single exposures varied from ten (10) to twenty (20) minutes. One must know well his apparatus, in order to give the proper dosage, in order to obtain the desired result.

From a review of all the cases, it is evident that practically all the cancers, commencing on the skin, can, in their early stages, be completely and permanently eradicated under the influence of the X-ray, in a simple and painless manner, without leaving a scar, and without any danger to the patient.

Much benefit can be expected in the advanced stages, by relieving pain, retarding the growth and prolonging life. The X-ray is of doubtful value in the treatment of cancers on the mucous membranes and primary growths of the mammry gland.

---

Editor *Journal-Record of Medicine*:—

Allow me to express my deep-felt sympathy with the address of Dr. H. Mc Hatton, of Macon, Ga., to the Young Men's Christian Association, reproduced in your September issue. But I would wish the liberty was granted me, benevolently, to extend the rational standpoint of the worthy Doctor in a direction which at first sight may appear extravagant, is really, however, only a summarizing of pertinent feelings. It is *the instruction of the light-o'-love-girls who make sexual relations a trade*. Off and anon doctors urge measures to shelter men from venereal contagion, but what of it, if not first the girls are protected? The doctors emphasize the fact that women can be sick, sexually, without knowing anything about it, while the men, upon the whole matter, cannot help exhibiting their condition, making it accessible to examination. But the girls ought to learn making this examination, to protect in the first instance, themselves. If they knew how, it is not likely that they would slight the advantage, and neglect the safeguard. The certain injury they inflict on themselves is out of proportion to the petty gain they may expect, and so their instruction puts a more efficient stop to the spread of venereal diseases than ever can be attained by measures calculated to shelter the men by direct measures.

I am aware of the fact that such a course of pathology dealt out to the *demi-monde* would in the beginning look rather awkward. But here I refer to the excellent words of Dr. Mc Hatton, in respect to the maxim, that "there is not enough accord between parents and children in our present type of life." For this holds true also with regard to the governed and the government; we must have more openness and frankness of dealing; the hide-and-seek game which has been played so long, has never benefitted society and must be exchanged for scientifically controlable demarches, the lofty character of which is above all scoffing and ridicule.

Dr. C. A. F. Lindorme.

